# Rarity-Weighted Richness: A Simple and Reliable Alternative to Integer Programming and Heuristic Algorithms for Minimum Set and Maximum Coverage Problems in Conservation Planning

**DOI:** 10.1371/journal.pone.0119905

**Published:** 2015-03-17

**Authors:** Fabio Albuquerque, Paul Beier

**Affiliations:** School of Forestry, Northern Arizona University, Flagstaff, Arizona, United States of America; Institut Pluridisciplinaire Hubert Curien, FRANCE

## Abstract

Here we report that prioritizing sites in order of rarity-weighted richness (RWR) is a simple, reliable way to identify sites that represent all species in the fewest number of sites (minimum set problem) or to identify sites that represent the largest number of species within a given number of sites (maximum coverage problem). We compared the number of species represented in sites prioritized by RWR to numbers of species represented in sites prioritized by the Zonation software package for 11 datasets in which the size of individual planning units (sites) ranged from <1 ha to 2,500 km^2^. On average, RWR solutions were more efficient than Zonation solutions. Integer programming remains the only guaranteed way find an optimal solution, and heuristic algorithms remain superior for conservation prioritizations that consider compactness and multiple near-optimal solutions in addition to species representation. But because RWR can be implemented easily and quickly in R or a spreadsheet, it is an attractive alternative to integer programming or heuristic algorithms in some conservation prioritization contexts.

## Introduction

In conservation planning, the minimum set problem [1] is to identify a set of sites (individual planning units) within a planning area that represent all conservation targets (typically species) in the fewest number of sites. It is closely related to the maximum coverage problem, which is to represent the largest number of species in a given number of sites. Both problems emphasize efficiency; the minimum set problem is appropriate for comprehensive long-term plans whereas maximum coverage is appropriate for short-term plans when resources are insufficient to meet all targets. A set of sites that satisfies or nearly satisfies a minimum set or maximum coverage problem is referred to as a “solution.” For over 30 years, conservation biologist have known that selecting sites in order of species richness provides poor solutions [2,3,4]. A good solution is not a set of richest sites, but a set of sites whose species assemblages complement each other and collectively capture the largest number of species. Three approaches are available to identify complementary sites that meet minimum set and maximum coverage problems.

Integer programming is the only method that can be guaranteed to identify optimum solutions [5]. However, there are 2 main reasons why integer programming is not widely used for these problems. First, for problems involving more than a few hundred sites, the number of possible combinations increases to astronomical numbers, and computing time can become prohibitively long. Second, integer programming often requires major oversimplification of the problem, such that integer programming provides an exact solution to an oversimplified problem statement [6].

Two heuristic algorithms are commonly used to solve minimum set and maximum coverage problems, namely the reverse stepwise search in the software package Zonation [7], and simulated annealing, typically implemented in the software package Marxan [8]. Although these algorithms produce solutions that cannot be guaranteed to be optimum, they consistently yield near-optimal solutions [6]. These algorithms are popular because of their computing speed, their ability to reflect goals other than species representation (e.g., compactness), and their ability to provide divergent solutions that provide flexibility for decision-makers. Nonetheless, implementing these heuristic algorithms requires considerable data pre-processing and model calibration, e.g., [7, 8]. It would be useful to have a simple, reliable alternative to integer programming and heuristic algorithms.

One intuitively appealing alternative is to assemble solutions in order of rarity-weighted richness, RWR. Williams et al. [9] proposed that the rarity value of a species can be characterized by the inverse of the number of sites or planning units in which it occurs. Thus if a species is found in only 1 site, the species would have the maximum rarity score of 1/1 = 1, and a species that occurs in 20 sites would have a rarity score of 1/20 = 0.05. Williams et al. also proposed that the rarity scores of all species in the site can be summed to yield a single RWR value for the site:

$$\sum_{1}^{n}\left(1/c_{i}\right)$$

where *c*_*i*_ is the number of sites occupied by species *i*, and the values are summed for the *n* species that occur in that site.

For one dataset of 426 vertebrate species mapped across 441 sites in Oregon, Csuti et al. [3] found that sites chosen in order of RWR represented species almost as effectively as optimum sets of sites identified by linear programming, and slightly more effectively than sites selected by simulated annealing. Csuti et al. suggested that the near-optimality, speed and simplicity of RWR made it suitable for prioritizing sites in large datasets. Despite this recommendation, RWR is rarely used in the academic literature, although it has occasionally been used to produce prioritization maps, most notably by Chaplin et al. [10], NatureServe [11], and California Department of Fish & Wildlife [12]. To the best of our knowledge, Csuti et al. provided the only comparison of RWR solutions to solutions produced by linear programming or heuristic algorithms. Perhaps additional positive results would increase the use of RWR to solve minimum set and maximum coverage problems.

In this paper we demonstrate that across 11 datasets, RWR represented species as efficiently as sites selected by the reverse stepwise algorithm used in the core-area version of Zonation [7]. Our goals are to call attention to RWR as a useful tool across a spectrum of spatial scales and to encourage wider consideration of RWR by conservation biologists.

Instead of (or in addition to) comparing RWR to the heuristic algorithm in Zonation, we could have compared RWR solutions to those produced by simulated annealing in Marxan. We chose to consider only Zonation because it is simpler to implement, and because the two packages produce similar results [13].

## Methods

### Datasets

We selected 7 plant datasets and 4 bird datasets that spanned 211 to 1456 species, 230 to > 85,000 sites, and site sizes from <1 ha to 2,500 km^2^ (Table 1). Datasets included both inventory and atlas data. Although atlas data include false absences, the atlas datasets for Western Europe, UK, and Spain are among the world’s most exhaustive atlas datasets on a consistent grid (Table 1 footnotes). In any event, false absences lead to conservative estimates of the effectiveness of shortcuts such as RWR [14].

**Table 1:** Datasets used to evaluate how well rarity-weighted richness selects sites that efficiently represent species.

Taxon, geographic area	# Sites	Size of each site	# Species	Type of dataset^1^
Plants, Sequoya-Kings Canyon National Park, USA^2^	545	< 1 ha	854	Inventory
Plants, Shenandoah National Park, USA^2^	351	< 1 ha	728	Inventory
Plants, Chiapas, Mexico [15]	230	< 1 ha	258	Inventory
Plants, Sierra Nevada, Spain [16]	595	4 ha	255	Inventory
Trees & shrubs, Spain^3^	85,474	1 km^2^	237	Atlas
Birds, Arizona, USA [17]	1,317	~6 km^2^	359	Inventory
Plants, UK^4^ [18]	2,242	100 km^2^	1,456	Atlas
Birds, Spain^5^ [19]	5,301	100 km^2^	294	Atlas
Birds, Florida, USA^6^	1,028	~196 km^2^	211	Atlas
Plants, Zimbabwe^7^	360	625 km^2^	1,338	Atlas
Birds, Western and Central Europe^8^ [20]	2,195	2,500 km^2^	424	Atlas

Datasets are listed in order of size of sites.

^1^In each inventory dataset, an attempt was made to inventory all species at each site. In each atlas dataset, each site was a grid cell, and the data consisted of all species records in the cell.

^2^ US National Park Service Inventory Products http://science.nature.nps.gov/im/inventory/veg/products.cfm (accessed 20 June 2014)

^3^ Ministry of Agriculture Food and Environment of Spain. Third National Forest Inventory; over 540,000 occurrences, 1997–2006. http://www.gbif.org/dataset/fab4c599-802a-4bfc-8a59-fc7515001bfa

^4^ over 9 million records.

^5^ 410,973 records.

^6^ Florida's breeding bird atlas: A collaborative study of Florida's birdlife. http://myfwc.com/bba (accessed 12 March 2014).

^7^ Data from http://www.gbif.org/dataset/1881d048-04f9-4bc2-b7c8-931d1659a354; 6316 records for Zimbabwe.

^8^ >100,000 records, covering areas west of Russia, Belarus, and Ukraine.

### Calculating number of species in sites prioritized in order of RWR

For each dataset, we calculated RWR for each site. We then accumulated the 5%, 10%, 15%,… 60% of sites with the highest RWR values. We stopped at 60% because RWR represented 99–100% of the total number of species in each dataset by this point. At each 5% percentile of prioritized sites, we calculated *S*, the number of species represented at least once in that set of sites.

### Calculating number of species in sites prioritized by Zonation

For each dataset, we used the basic core area formulation of the Zonation reserve selection software [7] to rank sites in terms of their importance to species representation. To produce a hierarchical prioritization of sites, Zonation starts with all sites tentatively ‘reserved’ and iteratively removes sites that are least needed to retain at least a few occurrences of each species. The algorithm minimizes the impact to the “worst-off” species, i.e., the species with the smallest remaining number of sites. Thus if the worst-off species occurs in only 4 sites, Zonation would not remove any of those 4 sites from the solution until it would be impossible to remove a site without causing 1 or more species to have fewer than 4 sites in the tentative solution. At that point Zonation would remove the site that causes the smallest number of species to be confined to 3 remaining sites. Sites receive a score between 0 and 1; values close to one indicate sites removed in the last state of the process whereas values close to 0 indicate sites removed early.

For the top 5%, 10%, 15%,… 60% of sites (as ranked by Zonation) we calculated *Z*, the number of species represented at least once. In many maximum coverage problems, *Z* is used as an estimate of the optimum or maximum number of species that can be represented in a given number of sites [4,7].

### Assessing RWR efficiency relative to Zonation and random selection of sites

We accumulated sites in random order and at each 5% increment (as above) we calculated the number of species represented at least once in the randomly selected sites. We repeated the random selection procedure 1,000 times and calculated *R* (the mean number of species across 1000 runs) and the 95% CI of *R* at each 5% increment.

Finally, we plotted *S*, *Z*, *R*, and the 95% CI of *R* against the percent of sites selected, and compared *S* and *Z* as follows. We considered *S-R* an measure of how much RWR improved on random selection of sites, *Z-R* a measure of how much Zonation improved on random selection of sites, and (*S-R*)/(*Z-R*) as an index of the improvement offered by RWR relative to the improvement offered by Zonation. The ratio (*S-R*)/(*Z-R*) is identical to the Species Accumulation Index, SAI, of Rodrigues & Brooks [14]. A value of 0.95 would indicate that RWR is 95% as effective as Zonation in improving on random selection of sites. A value of 1.0 would indicate that RWR is as effective as Zonation (and therefore a reasonable estimate of an optimal solution), and value of 1.05 would indicate that the RWR solution was 5% more effective than Zonation in improving on random selection of sites.

## Results

**Fig 1:**
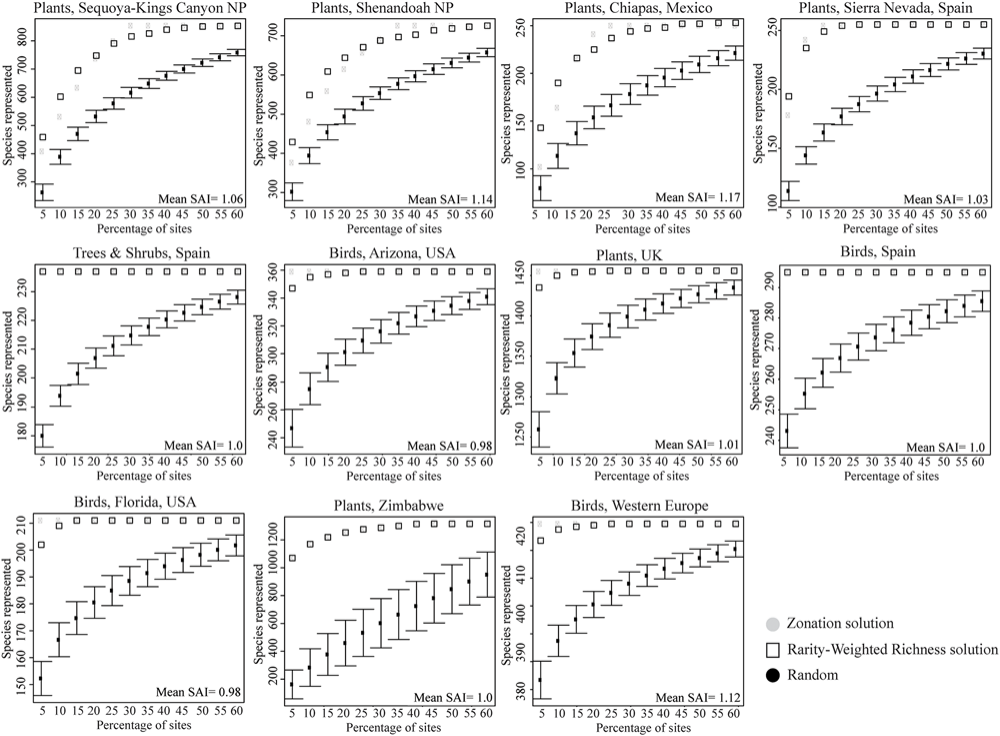
Number of species, *S*, represented at least once in sites selected in order of rarity-weighted richness and number of species, *Z*, represented at least once in sites selected by Zonation, compared to the number of species, *R*, represented in an equal number of randomly-selected sites. SAI is (*S-R*)/(*Z-R*), and describes the effectiveness of rarity-weighted richness to that of Zonation in terms of their ability to improve on random selection of sites.

For all 11 datasets and all percentages of sites prioritized, RWR and Zonation solutions represented many more species than the same number of randomly-selected sites (Fig. 1). On average across all datasets and percentages of sites prioritized, RWR solutions were 4% more effective than Zonation solutions in improving on random selection of sites (Table 2). In 43 instances, RWR solutions were better (closer to the true optimum) than Zonation (Fig. 1, Table 2). In 30 instances, Zonation provided better solutions than RWR. In the other 59 instances, RWR and Zonation solutions represented the same number of species.

**Table 2:** Species Accumulation Index, SAI, for sites prioritized in order of rarity-weighted richness (RWR) compared to sites prioritized by Zonation, for the 11 datasets described in Table 1.

Target (% of sites)	Plants, Sequoia-Kings NP	Plants, Shenandoah NP	Plants, Chiapas	Plants, Sierra Nevada, Spain	Trees & shrubs, Spain	Birds, Arizona	Plants, UK	Birds, Spain	Birds, Florida	Plants, Zimbabwe	Birds, Western Europe
5%	1.36	1.72	2.79	1.25	1.00	0.89	0.90	1.00	0.85	1.00	1.00
10%	1.51	1.78	1.51	0.93	1.00	0.95	0.96	1.00	0.96	1.00	1.00
15%	1.38	1.47	1.01	0.94	1.00	0.97	0.99	1.00	1.00	1.00	1.05
20%	1.05	1.24	0.82	1.00	1.00	0.98	1.00	1.00	1.00	1.00	1.07
25%	0.96	1.11	0.84	1.02	1.00	1.00	1.01	1.00	1.00	1.00	1.08
30%	0.84	0.97	0.92	1.02	1.00	1.00	1.02	1.00	1.00	1.00	1.10
35%	0.86	0.80	0.95	1.02	1.00	1.00	1.02	1.00	1.00	1.00	1.11
40%	0.92	0.82	0.96	1.02	1.00	1.00	1.02	1.00	1.00	1.00	1.14
45%	0.95	0.89	1.04	1.03	1.00	1.00	1.03	1.00	1.00	1.00	1.16
50%	0.98	0.93	1.05	1.03	1.00	1.00	1.03	1.00	1.00	1.00	1.19
55%	0.98	0.96	1.09	1.04	1.00	1.00	1.04	1.00	1.00	1.00	1.23
60%	0.99	1.00	1.10	1.04	1.00	1.00	1.05	1.00	1.00	1.00	1.28
Mean^1^	1.06	1.14	1.17	1.03	1.00	0.98	1.01	1.00	0.98	1.00	1.12

SAI describes how well RWR improves on random selection of sites relative to how well Zonation improves on random selection of sites, where the goal is to represent each species at least once. An SAI of 0.95 indicates that RWR is 95% as effective as Zonation in improving on random selection of sites, whereas an SAI of 1.05 indicates that RWR is 105% as effective as Zonation.

^1^ Grand mean is 1.04.

## Discussion

We conclude that the number of species in sites assembled in RWR rank order can be used as an estimate of the maximum number of species that can be represented in a given number of sites, or to estimate the minimum number of sites needed to represent all species. For the 11 datasets we analyzed, RWR solutions were about as efficient, and sometimes more efficient, than Zonation solutions. Similarly, Csuti et al. [3] found that RWR performed slightly better than simulated annealing. Because simulated annealing and Zonation are known to produce optimum or near-optimum solutions [6], RWR must also be producing optimum or near-optimum solutions. In all datasets, RWR represented 99–100% of the total number of species at most percentages of sites selected, leaving little room for superior solutions. Our results demonstrate that RWR’s ability to identify sites that efficiently represented Oregon vertebrates [3] was not a quirk of their particular taxonomic group, site size, or study area. RWR appears to be a simple, reliable alternative to heuristic algorithms for solving minimum set and maximum coverage problems.

The main advantage of RWR over linear programming and heuristic algorithms is that no specialized software, programming experience, or analytic skill is required. All calculations can be completed in R or in a spreadsheet. Although our evaluations used a uniform target of 1 occurrence per species, RWR can accommodate higher (more conservation-relevant) targets.

We acknowledge that RWR cannot replace linear programming, which remains the only procedure that can be guaranteed to find optimal solutions for these problems. RWR also cannot replace Marxan or Zonation, which (unlike RWR) can (a) include goals for compactness and connectivity, and (b) generate several near-optimal alternatives that provide flexible options for achieving conservation goals.

RWR is a simple metric that can be useful for at least 3 types of minimum set and maximum coverage problems. First, RWR is ideally suited to estimate *O*, one of the two benchmarks used in tests of surrogacy. Species Accumulation Index (SAI), the standard metric used to evaluate and compare surrogate strategies [14], compares *S*, the number of species represented at least once in a specified number of sites prioritized by the surrogate to two benchmarks: *R*, the mean number of species represented at least once in the same number of randomly selected sites and *O*, the largest number of species that can be represented at least once in that number of sites. As mentioned in the previous paragraph, real-world site selection often involves compactness, connectivity, species-specific levels of representation, and diversity of near-optimal solutions. However, these considerations are ignored in surrogacy tests, for which an arbitrary 1 occurrence per species provides a simple, repeatable criterion for representing each species. As demonstrated here, RWR can simplify the calculation of *O*, and thus streamline the testing of surrogates.

Second, RWR can provide a first approximation of priority areas for meeting the goal of representing all species. For example, Chaplin et al. [10] and CDFW [12] used RWR to map areas that would efficiently represent rare species in the coterminous USA, and in California, respectively. In both cases, the authors felt that RWR made good use of occurrence data that were too sparse to support reliable species distribution models. Third, RWR can be used to assess the efficiency of a set of existing or proposed protected sites. If most high-RWR sites lie outside of protected sites, planners could conduct further analyses to determine if particular species in the unprotected RWR hotspots are in need of additional protection.
